# Supplementary material for: How adaptive plasticity evolves when selected against
Source: PLoS Comput Biol. 2019 Mar 8;15(3):e1006260. doi: 10.1371/journal.pcbi.1006260 (PMC6426268; doi:10.1371/journal.pcbi.1006260)
Supplement: S1 Appendix — Numerical calculations of the expected long-term fitness costs of adaptively plastic and adaptive tracking populations. (PDF) [file pcbi.1006260.s001.pdf]

S1 Appendix: Cost of Plasticity and Tracking

In order to compare the long-term advantage of plasticity over tracking, we calculate their respective fitness costs. The average cost of plastic strategies can be easily calculated as λb, where b is the slope of the optimal reaction norm. (see section Evaluation of Fitness in main paper). The cost of tracking will instead be determined by the average fitness during the tracking phase multiplied by the proportion of evolutionary time the population spends tracking (as opposed than on local optima). The latter term will be determined by the amount of time spent tracking after each environmental change relative to the time between environmental changes, so that:

$$LoF = f^{\bar{E_i}} \frac{t_{tr}}{K} \tag{1}$$

Where $f^{\bar{E_i}}$ is the average decrease in fitness while tracking and $1/K$ the frequency of environmental changes and t_{tr} the time spent tracking after each environmental change. In order to calculate those terms, we assume that mutations which provide a fitness advantage are immediately fixed, while those that do not are immediately lost. This allows us to calculate the average fitness during tracking as $f^{\bar{E_i}} = \frac{\bar{f_{t_0}} + \bar{f_{t_{tr}}}}{2}$, where $\bar{f_{t_0}}$ is the the average fitness loss immediately after an environmental change and $\bar{f_{t_{tr}}}$is the average fitness loss at the end of the tracking period. The time required to reach the new short-term optimal phenotype t_{ad} will be:

$$t_{ad} = \frac{\bar{\Delta p}}{\bar{\sigma}/2 + \bar{c}\bar{\sigma}/2} \tag{2}$$

Where $\bar{\Delta p}$ is the average distance between phenotypic targets after each environmental change, $\bar{c}$ the average value of environmental cues and $\bar{\sigma}$ the mutational variance of intercept and slope. If $t_{ad} \leq K$, the tracking population will be able to reach the new optimum in t_{ad} evolutionary steps, so that $t_{tr} = t_{ad}$. If instead $t_{ad} > K$, the population will only be able to adapt to each optima for K evolutionary steps, so that $t_{tr} = K$. The overall value of t_{tr} can thus be calculated as the minimum of either t_{ad} or K.
While the tracking population also evolves by changing its slope, the average direction of selection will be equally likely to be positive or negative after each environmental change, so that the average slope values remain zero for both the tracking and optima phases. As such, we do not include the cost of plasticity in the calculations for the cost of adaptive tracking.

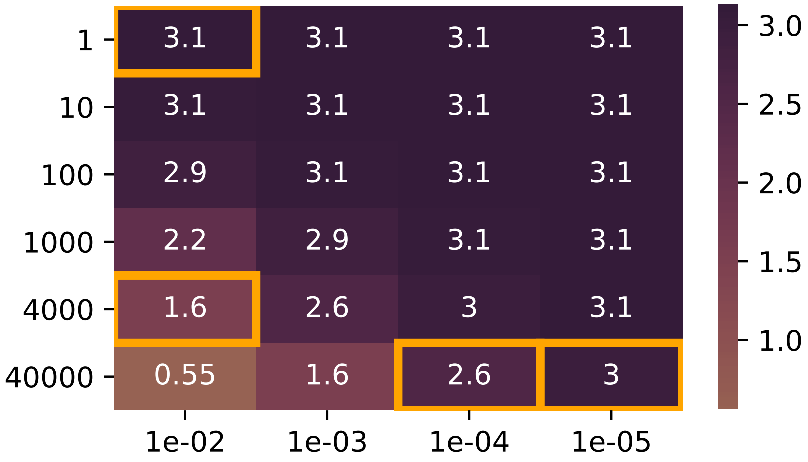

Figure A: **Long-term fitness cost of adaptive tracking relative to adaptive plasticity.** Rows correspond to values of K (generations before each environmental change) and columns to average mutation rates. Values within each cell show the cost of tracking compared to that of plasticity, on a log10 scale.

As we show in figure A, adaptive plasticity incurs lower long-term fitness costs than adaptive tracking over all parameter values tested in our simulations.
