# Supplementary figures and images for: How adaptive plasticity evolves when selected against
Source: PLoS Comput Biol. 2019 Mar 8;15(3):e1006260. doi: 10.1371/journal.pcbi.1006260 (PMC6426268; doi:10.1371/journal.pcbi.1006260)

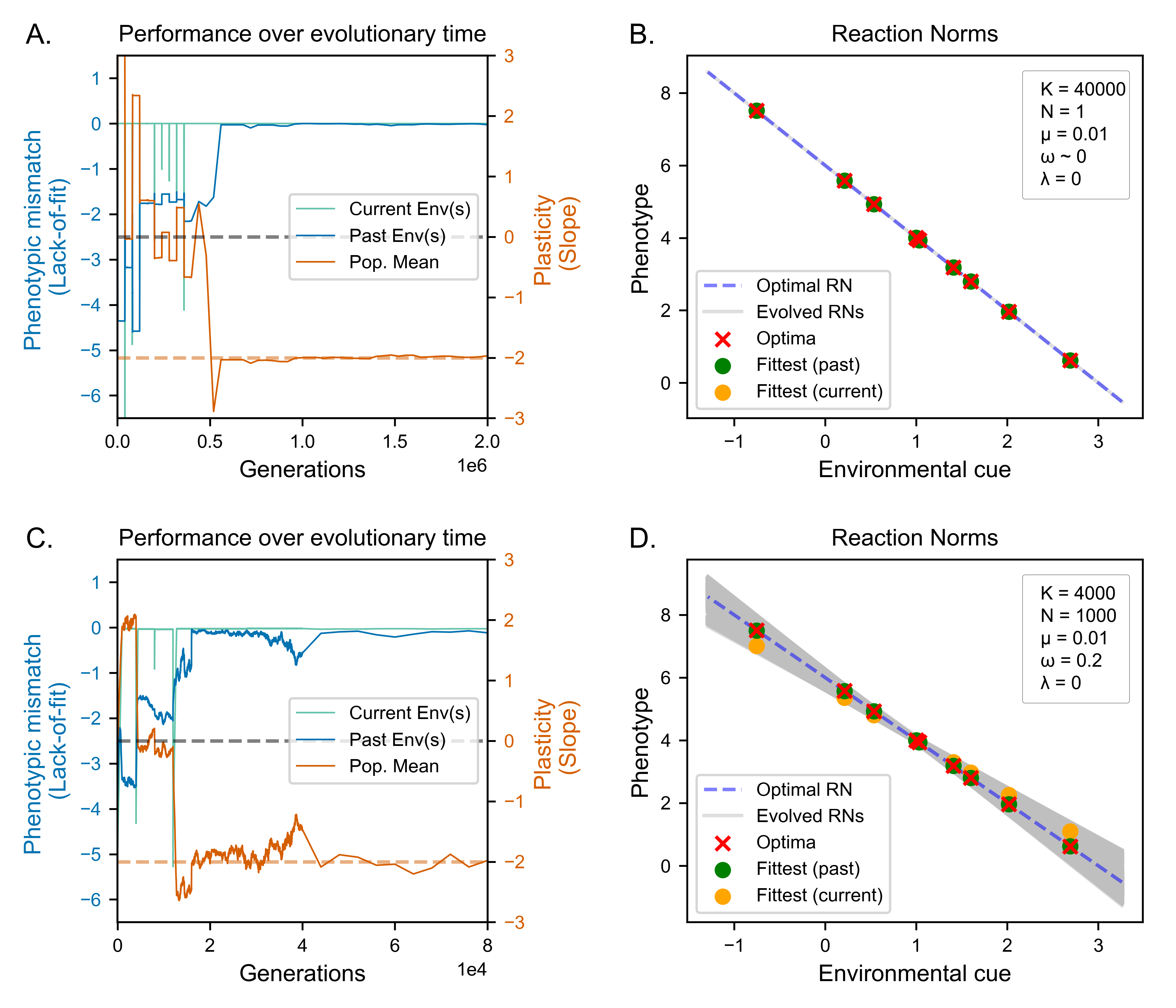

Supplement: S1 Fig — Evolutionary trajectories and evolved plasticity in the absence of cost of plasticity for SSWM (A and B) and population models (C and D). (TIF) [file pcbi.1006260.s002.tif]
